# Effectiveness of the Chronic Care Model in Type 2 Diabetes Management in a Community Health Service Center in China: A Group Randomized Experimental Study

**DOI:** 10.1155/2019/6516581

**Published:** 2019-01-03

**Authors:** Jing-Xia Kong, Lin Zhu, Hong-Mei Wang, Ying Li, An-Ying Guo, Chao Gao, Yan-Yao Miao, Ting Wang, Xiao-Yang Lu, Hong-Hong Zhu, Donald L. Patrick

**Affiliations:** ^1^Department of Social Medicine and Department of Pharmacy of the First Affiliated Hospital, Zhejiang University School of Medicine, Hangzhou, Zhejiang Province, China; ^2^Department of Investment and Insurance, Zhejiang Financial College, Hangzhou, Zhejiang Province, China; ^3^Department of Social Medicine, Zhejiang University School of Medicine, Hangzhou, Zhejiang Province, China; ^4^Department of Pharmacy of the First Affiliated Hospital, Zhejiang University School of Medicine, Hangzhou, Zhejiang Province, China; ^5^Preventive Medicine Institute, Louisiana, MO 63353, USA; ^6^Department of Health Services, University of Washington, H670 Health Sciences Building, Box 357660, Seattle, WA 98195-7660, USA

## Abstract

**Objective:**

The Chronic Care Model, based on core elements of team-centered care in chronic diseases, has widely been accepted. This study was aimed at evaluating the effectiveness of the Chronic Care Model in type 2 diabetes management.

**Methods:**

A group randomized experimental study was conducted. Twelve communities of the Zhaohui Community Health Service Center in Hangzhou, China, were randomly assigned into an intervention group (*n* = 6) receiving the Chronic Care Model-based intervention and a control group (*n* = 6) receiving conventional care. A total of three hundred patients, twenty-five for each community, aged ≥18 years with type 2 diabetes for at least 1-year duration, were recruited. Data of health behaviors, clinical outcomes, and health-related quality of life (Short-Form 36-item questionnaire) were collected before and after a 9-month intervention and analyzed using descriptive statistics, *t*-test, chi-square test, binary logistic regression, and linear mixed regression. A total of 258 patients (134 in intervention and 124 in control) who completed the baseline and follow-up evaluations and the entire intervention were included in the final analyses.

**Results:**

Health behaviors such as drinking habit (OR = 0.07, 95% CI: 0.01, 0.75), physical activity (OR = 2.92, 95% CI: 1.18, 7.25), and diet habit (OR = 4.30, 95% CI: 1.49, 12.43) were improved. The intervention group had a remarkable reduction in glycated hemoglobin (from 7.17% to 6.60%, *P* < 0.001). The quality of life score changes of the role limitation due to physical problems (mean = 9.97, 95% CI: 3.33, 16.60), social functioning (mean = 6.50, 95% CI: 2.37, 10.64), role limitation due to emotional problems (mean = 8.06, 95% CI: 2.15, 13.96), and physical component summary score (mean = 3.31, 95% CI: 1.22, 5.39) were improved in the intervention group compared to the control group.

**Conclusion:**

The Chronic Care Model-based intervention helped improve some health behaviors, clinical outcomes, and quality of life of type 2 diabetes patients in China in a short term.

## 1. Introduction

Diabetes is one of the most common metabolic disorders in the world and its prevalence in adults was increasing in the last decades [1, 2]. The International Diabetes Federation (IDF), Diabetes Atlas, shows that there are 425 million people with diabetes mellitus (DM) with a prevalence rate of 8.8% in adults [3]. Urbanization has driven dramatic changes in lifestyle particularly in developing countries, which results in a high incidence of obesity and related chronic diseases including diabetes. As the largest developing country in the world, China experienced a sharp increase in the incidence of type 2 diabetes mellitus (T2DM) in the past years. Recent studies have shown that the prevalence of diabetes in China has reached nearly 11% among Chinese adults, which is much higher than the world average rate [3, 4]. China has become the top country with the largest number of people with diabetes in the world [3].

The main risk factors of T2DM include alcohol consumption, physical activity, diet, obesity, weight, blood glucose, serum lipid, and blood pressure. These factors play important roles in diabetes control, the development of diabetic complications, and the patients' quality of life [5–7]. Health-related quality of life (HRQoL) is a subjective assessment of health status, including general health, physical, emotional, cognitive, and role functioning, as well as social well-being and functioning, which received increasing attention from health professionals and general public. HRQoL, however, can be used as a useful health outcome evaluation tool for chronic diseases such as T2DM. HRQoL is lower among adults with T2DM compared with those without [8–12]. Improvements in HRQoL among patients with T2DM had been demonstrated with the initiation of some antidiabetic therapies in several previous studies [13, 14] while few studies up to date have reported the relationship between T2DM management and HRQoL.

Management of patients with T2DM is a growing public health concern because of its increased incidence and costs and complexity of care. Researchers and practitioners are challenged to find efficient and effective ways to improve diabetes management. The Chronic Care Model (CCM), originating from a systematic research program in the United States, provides a blueprint for chronic disease management [15–18]. The CCM comprises six components that are hypothesized to affect functional and clinical outcomes associated with disease management. These six components are (1) health system—organization of health care (providing leadership for securing resources and removing barriers to care); (2) self-management support (facilitating skill-based learning and patient empowerment); (3) decision support (providing guidance for implementing evidence-based care); (4) delivery system design (coordinating care processes); (5) clinical information systems (tracking progress through reporting outcomes to patients and providers); and (6) community resources and policies (sustaining care by using community-based resources and public health policy) [16, 17]. A central element of the CCM is the team-centered care approach, which facilitates and produces effective interactions between proactive primary care practice teams and empowers patients with the aim to improve processes and outcomes in patients with chronic illnesses. Changes in multiple areas are preferred in order to improve the quality and outcomes of diabetes care considerably. A meta-analysis study showed that three to four component interventions attained stronger effect estimates than two component interventions did [19]. The whole model or at least some of its elements has been increasingly accepted and implemented in many countries [20–22]. The effectiveness of this model in community diabetes management has also been demonstrated in studies including systematic reviews and randomized controlled trials [23–25]. Studies with the CCM approach in China, however, are limited. Most community-based interventions for diabetes management in China mainly focus on patient education, team management, and self-management support [26–28]. Only one study using cross-sectional study design addressed the relationship between compliance with the CCM in community health centers and self-management behaviors, glycemic control, and finally the utilization of community health centers for monitoring and treating diabetes [29]. As far as we are aware, few studies in China have evaluated the relationships between the application of the CCM and objective health indicators such as glycosylated hemoglobin A1c (HbA1c) and subjective health indicators such as HRQoL using randomized controlled trials in community health service centers.

This study was therefore aimed at assessing the effects of the CCM-based intervention on T2DM Management Program in Hangzhou, China, which adopted five components of the CCM framework. We hypothesized that primary outcomes about health behaviors (smoking, drinking, physical activity, and diet habit) and secondary outcomes about clinical outcomes (glycemic value, blood pressure, and lipid level) and HRQoL would be improved in T2DM patients who received the CCM-based intervention in community health service centers.

## 2. Methods

### 2.1. Study Design and Subjects

A 9-month group-based randomized experimental study was designed and conducted to evaluate the effectiveness of the CCM-based intervention in T2DM management at the Zhaohui Community Health Service Center in Hangzhou, Zhejiang province, China. The community health service center covers 12 communities with a geographic area of 3.03 square kilometers. Twelve physician teams from the Department of Chronic Disease Management in this center serve for the 12 communities, respectively. These 12 communities were randomly assigned into an intervention group that received the CCM-based care with components including health system, self-management support, decision support, delivery system design, and clinical information system and a control group that received conventional care. Six communities were included in the intervention group and six in the control group. All physicians involved in the study received trainings on community diabetes management guidelines and those in the intervention group were required to complete additional training on the CCM including knowledge, technical, and related tools. The initial sample size was calculated on the basis of an absolute difference in HbA1c of 0.4% [30]. With a two-tailed power of 80% at a 0.05 alpha level, assuming an intraclass correlation coefficient (ICC) of 0.002 and taking into account a correction factor for the clustered design, it was calculated that 6 × 21 (126) patients would be needed in each group to detect the difference. Allowing for 15% drop out rate, a total of 290 patients need to be included. A total of 300 patients were finally recruited from the 12 communities, with 25 randomly sampled from eligible patients in each community tracked by the chronic disease management information system. Inclusion criteria were an age of 18 years or older and at least 1-year duration of T2DM. Patients with difficulties to a self-administered survey due to cognitive or reading issues were excluded from the study. Participants completed questionnaires of demographic and clinical information, clinical laboratory tests, and health-related quality of life at baseline in September, 2009. After 9 months' intervention, the same questionnaires and clinical tests were administered again in June, 2010.

### 2.2. Measures

#### 2.2.1. Control Group (Conventional Care)

The control group received conventional follow-up, which was applied every three months by their responsible physicians through office visits, home visits, and telephone calls. Changes in lifestyle, diabetes control, compliance to treatment, side effects of drugs, and target organ damage for each patient were examined and general care guidance was given.

#### 2.2.2. Intervention Group (CCM)

The intervention group received the five components CCM-based intervention. The component of community resources and policies was not included in the intervention due to poor coordinated care between primary and secondary care at the time of this study.

*(1) Health System: Stimulation of Policy-Making*. Physicians were required to enhance patients' awareness of chronic disease management and encourage patient initiative through pamphlets and face-to-face communication. Additional subsidies were given to the physicians every month throughout the intervention process by the community health service center. Appropriate supervision and evaluation procedures were also followed.

*(2) Self-Management Support*. Self-management support strategies included goals setting, planning, doing, checking, and assessing. The physicians helped their patients set goals and made monthly self-management plans. The patients filled in a self-management checklist semimonthly and reported it to their physicians. At the end of each month, the physicians checked each patient's condition and helped the patient plan for the next month.

*(3) Decision Support*. Decision support included implementation of the clinical guidelines, continuous medical education, and feedback of baseline medical records. The physicians had clinical guidelines training and continuous medical education provided by the community health service center. The results of patients' baseline survey were reported to their responsible physicians to help in better understanding care provision.

*(4) Delivery System Design*. Each team included a responsible physician, a health manager, and a public health assistant. Clear assignment of roles and tasks within the team played an important role in the component. The primary duties of each team in this group were to help the patients self-manage their diseases (by the health manager), monthly follow-up (by the responsible physician), and respond to concerns of patients and other regular tasks (by the team together).

*(5) Clinical Information System*. The chronic disease management information system was used in the community health service center to provide population-based care for patients with hypertension, diabetes, and cancer including tracking, disease management, and assessment. The system could share data between the community health service center and belonging stations, also between primary and tertiary care. Patients' data were regularly collected to facilitate efficient and effective care. The physicians in the intervention group got reminders of monthly follow-up from the tracking system and were required to document feedback information timely. The physicians in the control group got reminders every three months.

### 2.3. Outcomes

Health behaviors were defined as the primary outcomes in our study. Health behaviors in this study included frequent smoker who smoked one or more cigarettes a day (yes or no), frequent drinker who drank at least once a week, with an average intake of 25 g pure alcohol per day or above (yes or no), physical activity (≥1 time(s)/week, none), and self-reported light diet defined as low-fat diet (yes or no). Clinical outcomes and HRQoL were defined as secondary outcomes in our study. Clinical outcomes included body mass index (BMI), waist circumference (WC), fasting blood glucose (FBG), HbA1c, blood pressure, and serum lipid. HRQoL was assessed with a Chinese (mainland) version of the Short Form 36 (SF-36). The SF-36 is a validated [31, 32] 36-item instrument including eight scales: physical functioning (PF), role limitations due to physical problems (RP), bodily pain (BP), general health (GH), vitality (VT), social functioning (SF), role limitations due to emotional problems (RE), and mental health (MH). The physical component summary (PCS) and mental component summary (MCS) scores can be calculated on the basis of these eight separate scales. The scale scores range from 0 to 100, with higher scores indicating a better health status. The PCS and MCS have been standardized on the basis of a normative Chinese general population data set, with a mean of 50 (Standard Deviation, SD, of 10) [31]. Higher summary scores indicate a better self-reported HRQoL as well.

### 2.4. Data Collection

At the baseline, general demographic and clinical information including age, gender, marital status, educational level, household income, employment status, diabetes duration, diabetes medication and diagnosis of other chronic disease, health behaviors, and HRQoL were collected by self-administered questionnaires. Clinical outcomes were measured by the responsible physicians or the clinical test department in this community health center. All the survey and lab tests were repeated after 9 months.

### 2.5. Statistical Analysis

Baseline general characteristics of age, gender, marital status, educational level, household income, employment status, diabetes duration, diabetes medication, and diagnosis of other chronic disease were described using frequency, means, ratios, and SDs. Independent sample *t*-tests for continuous variables and chi-square tests for categorical variables were used to examine differences between the two groups in demographic characteristics, clinical outcomes, health behaviors, and scores of the SF-36. Paired *t*-tests for continuous data and chi-square test for categorical data were used to determine within-group differences between baseline and follow-up. Binary logistic regression was used to analyze the associations between health behaviors (yes/no) and independent variables. Independent variables included group (control/intervention), health behaviors (yes/no) at baseline, and individual characteristics including age, gender (male/female), educational level (elementary school or below/junior middle school or higher), household income (<¥60000/≥¥60000), marital status (married or cohabiting/single, widowed, or divorced), employment status (employed or housework/retirement), diabetes duration (1-5 years/≥5 years), diabetes medication (insulin therapy or not), and diagnosis of other chronic disease (yes/no or unknown), respectively. The levels of association between independent variables and dependent variables were expressed as odds ratios (OR) and 95% confidence intervals (CI). Linear mixed regression was used to compare the change in clinical outcomes and HRQoL from baseline to follow-up between the two study groups. The clusters of community were fitted as random effects. The effects of time and the intervention were included as fixed effects, respectively, in the models. The effects of correlates including age, gender, educational level, household income, marital status, employment status, diabetes duration, diabetes medication, and diagnosis of other chronic disease were adjusted in all models. Additional missing values analysis was conducted for clinical outcomes using Little's test and missing values were imputed using the Expectation-Maximization (EM) method based on maximum likelihood estimates [33]. SPSS 20 for Windows was used for data analysis.

## 3. Results

**Figure 1:**
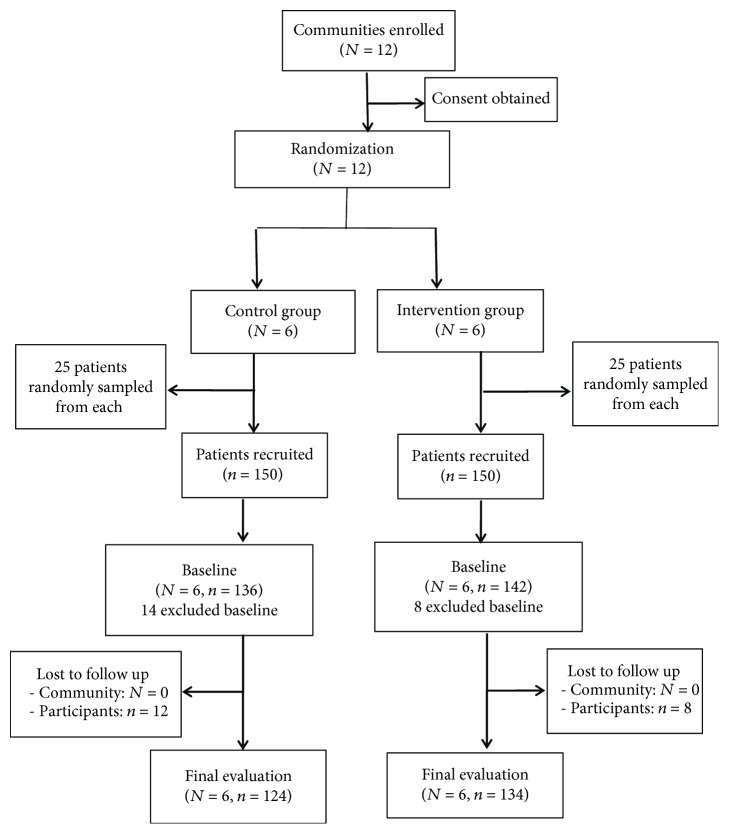
Flow diagram for participant recruitment from 12 communities in the Zhaohui Community Health Service Center for a 9-month group randomized experimental study in China, 2009-2010.

Of the total 300 recruited subjects, 278 (142 in the intervention group and 136 in the control group) completed the baseline evaluation and 258 (134 in the intervention group and 124 in the control group) completed the final evaluation after follow-up. Per-protocol analysis was performed and reported in major outcomes, in which only 258 patients completed the baseline and follow-up evaluations, and the entire intervention was included in the final analyses.  Figure 1 shows the participants flow chart in the study.

Baseline sociodemographic characteristics, health behaviors, clinical outcomes, and scores of the SF-36 were comparable for intervention and control groups except for the marital status, diabetes duration, diagnosis of other chronic diseases, the percentage of light diet, and the SF and RE scores (Table 1).

### 3.1. Primary Outcomes: Health Behaviors

As shown in Table 2, patients in the intervention group were less likely to be frequent drinkers (OR = 0.07, 95% CI: 0.01, 0.75), while they were more likely to follow frequent exercise (OR = 2.92, 95% CI: 1.18, 7.25) and light diet (OR = 4.30, 95% CI: 1.49, 12.43) than patients in the control group.

### 3.2. Secondary Outcomes: Clinical Outcomes and HRQoL

#### 3.2.1. Clinical Outcomes

The clinical outcomes were presented by the changes of objective indicators between baseline and postintervention by both within-group and between-groups.

As shown in Table 3, both the intervention and control groups had statistically lower FBG and diastolic blood pressure (DBP) after 9 months. The intervention group also had a remarkable reduction in HbA1c (7.17% to 6.60%, *P* < 0.001) and waist circumference (83.14 cm to 79.66 cm, *P* < 0.001), whereas there was no statistical difference in these two indicators in the control group. Patients in the intervention group reported no statistical difference in BMI post intervention while those in the control group had higher BMI (24.18 to 24.69, *P* = 0.004). Differences in systolic blood pressure (SBP), total cholesterol, high-density lipoprotein (HDL) cholesterol, low-density lipoprotein (LDL) cholesterol, and triglyceride in either group were not significant.

The between-group effects on the clinical indicators were estimated using linear mixed regression. When the effect of group was adjusted for patients' age, gender, marital status, educational level, household income, employment status, diabetes duration, diabetes medication, and diagnosis of other chronic disease, no statistically significant between-group intervention effects were observed on clinical outcomes. Missing value analysis suggested that data are missing completely at random (Little's test *χ*^2^ = 33.27, *P* = 0.31). When missing values were imputed using the Expectation-Maximization (EM) method based on maximum likelihood estimates, significant between-group intervention effect was observed in HbA1c (*P* = 0.001).

#### 3.2.2. HRQoL

Changes of HRQoL between baseline and follow-up were further examined by both within-group and between-groups (Table 4).

In the intervention group, scores of four scales of the SF-36 instrument significantly increased after the 9 months' CCM-based intervention (PF: 67.90 to 76.45, *P* < 0.001; RP: 75.00 to 91.23, *P* < 0.001; RE: 85.82 to 96.27, *P* < 0.001; and PCS: 48.34 to 52.31, *P* < 0.001), while the scores of VT (46.26 to 42.17, *P* = 0.002) and MCS (43.86 to 40.98, *P* < 0.001) significantly decreased. No changes occurred in the scores of BP, GH, SF, and MH scales after the intervention. In the control group, at the end of the follow-up, patients reported higher scores in the scales of VT (46.61 to 50.08, *P* = 0.03), SF (76.11 to 80.85, *P* = 0.02), RE (74.73 to 84.14, *P* = 0.04), and MCS (42.86 to 44.90, *P* = 0.02) and lower scores of BP (82.97 to 75.81, *P* < 0.001) and GH (44.85 to 40.32, *P* < 0.001) compared with those at the baseline. No changes were observed in the scores of PF, RP, MH, and PCS.

As to the between-group changes, after adjustment for patients' age, gender, marital status, educational level, household income, employment status, diabetes duration, diabetes medication, and diagnosis of other chronic disease, the differences remained statistically significant in RP (mean = 9.97, 95% CI: 3.33, 16.60), VT (mean = −5.43, 95% CI: -7.98, -2.89), SF (mean = 6.50, 95% CI: 2.37, 10.64), RE (mean = 8.06, 95% CI: 2.15, 13.96), and PCS (mean = 3.31, 95% CI: 1.22, 5.39). The score changes of RP, SF, RE, and PCS in the intervention group were higher than those in the control group, while the score changes of VT in the intervention group were lower than those in the control group.

## 4. Discussion

This is a group randomized experimental study that found significant improvements in lifestyle such as drinking habit, physical activity, and eating habit and parallel improvements in some clinical outcomes and HRQoL after the 9-month CCM-based intervention. These results were in line with the findings of previous RCTs using the CCM, where clinical and behavior outcomes in diabetes care were improved [23–25]. In China, the patients were more likely to use tertiary hospitals, for many patients believe that the quality of care provided by community health service is low [34]. In recent years, the government put more and more priorities on community health service because of its first contact, continuousness, cost-effectiveness, and convenience. As a comprehensive approach, the CCM might be a comprehensive approach to improve chronic care and health outcomes for patients with diabetes and further increase utilization of community health service for monitoring and treating diabetes and other chronic diseases. After the CCM-based intervention, significant improvements were achieved in lifestyle changes, especially in healthy diet habit. These results showed that the CCM-based intervention used in this study appeared to be an effective means of stimulating and maintaining lifestyle changes. Lifestyles such as physical activity and light diet would help control diabetes. As shown in other studies, compliance with lifestyle modification is more difficult to achieve than drug compliance. Patients' motivation for lifestyle change and maintenance will depend on “tools” and belief that the changes are achievable and worthwhile [35, 36]. The CCM framework in this study provided such a “tool” to enhance patients' self-management awareness and self-monitoring capabilities through patient-physician cooperation during comprehensive implementation of self-management support, care coordination, and clinical information tracking. It is consistent with prior study that greater motivation from attending physicians was associated with better performance of patient self-management [37].

Aside from the improvement in lifestyle modification, additional improvements occurred in clinical outcomes of waist circumference, FBG, HbA1c, and DBP after the CCM-based intervention, while no statistical difference in waist circumference and HbA1c was observed in the control group. When between-group comparison was explored, significant intervention effect was observed in HbA1c with missing value imputation. It was consistent with prior studies reporting that the lifestyle improvement contributed to good glycemic control [38, 39]. As a more stable indicator of monitoring glycemic control than FBG, HbA1c is also an important indicator of the risk of diabetic complications. Stratton et al. [40] found that the risk of the microvascular and macrovascular complications of T2DM was strongly associated with hyperglycemia as measured by HbA1c. Every 1% reduction in HbA1c was associated with a 37% decrease in risk for microvascular complications and a 21% reduction in the risk of any end point or death related to diabetes.

Our findings are consistent with other studies suggesting that the CCM is related to the control of HbA1c and blood pressure [41–45]. For example, Parchman and colleagues found that HbA1c scores were the lowest in diabetic patients whose primary care conformed the most to the CCM. Other studies also found that glucose control was associated with the extent to which the care delivered was consistent with the CCM and patient self-care behaviors such as diet and exercise [46, 47]. Primary care practices were complex adaptive systems, which were a diverse collection of agents that had the capacity to adapt or coevolve with their environment and were highly interconnected or interdependent [48]. It is possible that the CCM describes characteristics of the environment, which agents in the services, including patients, interacting, enhancing the relationship between the community and patients, improving the efficiency of community management, and resulting in outcomes such as glucose control.

HRQoL of patients with T2DM assessed by the SF-36 instrument improved in multiple domains in our study. After adjusting for correlates [49], statistical increase remained strong in the RP, SF, RE, and PCS scores of the SF-36 instrument, implying that the CCM-based intervention was effective to improve functioning for patients with T2DM, especially in physical health. The findings are consistent with prior study suggesting that educational interventions, low-calorie diet, and exercise produced significant improvements in quality of life especially in physical functioning [50, 51]. It was likely educational interventions improved patients' self-management awareness and capabilities, which led to self-care behaviors and changes in HRQoL [52, 53]. Our study showed that the VT and MCS scores significantly decreased after a 9-month CCM-based intervention and the decreased score change of the VT scores between the intervention and control groups reached statistical significance while the change of the MCS scores between two groups did not. These potential adverse effects in HRQoL of the CCM-based intervention are supported by other studies [54–56]. Patient education might lead to a higher awareness of the disease, which may be associated with higher levels of anxiety or lower HRQoL [54, 55]. Exercise consumes a lot of energy and may have increased muscle or joint pain [56].

Our findings suggest that the implementation of the CCM in T2DM management in community health service could be beneficial. The core element of the CCM is the interaction between physicians and patients, which benefits patients' self-management awareness and skills development which further improves patients' health outcomes. At present, patients in China were more likely to use tertiary hospitals when they need medical help because community health services were thought less effective [57, 58]. It is very important for community health services in China to develop or adapt effective disease management models in order to take good care of millions of diabetic patients due to limited numbers of physicians in the hospitals.

This study has some limitations. First, the representativeness of our study might be compromised due to not a big sample size and a small percentage of missing though the missing value imputation provided some additional results. Second, a 9-month study duration may not be long enough to detect some significant effects or to observe some improvements between the intervention and control groups. Whether improvements are sustainable maybe needs to be examined in future studies. Third, there were a small number of dropouts due to refusal or inaccessibility after the intervention; however, no data were available for us to do intention-to-treat analysis. Fourth, neither the patients nor the physicians were blinded to the treatment assignment because of differences in care between the intervention and control groups, but there was no evidence that patients in the intervention group were more likely to report positive outcomes than those in the control group and vice versa; and there was no evidence that physicians might tend to treat all patients in the intervention group more carefully than those in the control group due to such a big patient population in China. Our data collectors and analysts were blind to group assignment. Overestimation of the effect size by physicians should be random though it might be a concern. Finally, only five components were applied due to poor coordinated care between primary and secondary care at the time of this study. With the development of referral system nowadays, the component of community resources and policies could be further explored.

In summary, this group-randomized experimental study found that, after a 9-month CCM-based intervention to T2DM patients, improvements in lifestyle changes such as drinking habit, physical activity, and healthy diet, clinical outcomes of HbA1c, and evidences in physical health and social functioning improvement of HRQoL using the SF-36 instrument were found in the intervention group compared to the control group. The CCM is a comprehensive approach that helps improve chronic care and health outcomes of patients with diabetes in the community health service center in China in a short term. Long-term intervention with all six components of the CCM and new techniques such as web-based self-management could be further examined in more community health service centers in China. It is very important for community health services in China to develop or adapt effective disease management models in order to take good care of millions of diabetic patients due to limited numbers of physicians in the hospitals.

## Figures and Tables

**(a):** 

Characteristics		Intervention (*N* = 134)	Control (*N* = 124)	*P* value
Age (years)		69.12 ± 10.54	71.48 ± 8.79	0.05
Gender	Male	56 (41.8)	54 (43.5)	0.78
	Female	78 (58.2)	70 (56.5)	
Marital status	Single or divorced	2 (1.5)	4 (3.2)	0.01^∗^
	Married or cohabiting	103 (76.9)	109 (87.9)	
	Widowed	29 (21.6)	11 (8.9)	
Educational level	Illiterate	10 (7.5)	16 (12.9)	0.42
	Elementary school	44 (32.8)	35 (28.2)	
	Junior middle school	44 (32.8)	47 (37.9)	
	High school	23 (17.2)	18 (14.5)	
	College or university	13 (9.7)	8 (6.5)	
Household incomes (Yuan, $1US = 6.7 Yuan)	<60000	110 (82.1)	100 (81.5)	0.33
	≥60000, <200000	24 (17.9)	21 (16.9)	
	≥200000	0 (0)	2 (1.6)	
Employment status	Employee/self-employed	6 (4.5)	3 (2.4)	0.31
	Retirement	125 (94.0)	116 (93.5)	
	Housework	2 (1.5)	5 (4.0)	
Diabetes duration (years)	1–5	38 (28.4%)	21 (16.9%)	0.03^∗^
	≥5	96 (71.6%)	103 (83.1%)	
Diabetes medication	None	13 (9.7%)	14 (11.3%)	0.31
	Oral agents	102 (76.1%)	97 (78.2%)	
	Insulin	13 (9.7%)	5 (4.0%)	
	Oral agents and insulin	6 (4.5%)	8 (6.5%)	
Diagnosis of other chronic disease	Yes	13 (9.7%)	33 (26.6%)	<0.001^∗∗^
	No	104 (77.6%)	86 (69.4%)	
	Unknown	17 (12.7%)	5 (4.0%)	
*Health behaviors*				
Frequent smoker	Yes	9 (6.7)	6 (4.8)	0.52
	No	125 (93.3)	118 (93.5)	
Frequent drinker	Yes	10 (7.5)	10 (8.1)	0.86
	No	124 (92.5)	114 (91.9)	
Frequent Exercise	Yes	110 (82.7)	95 (76.6)	0.22
	No	23 (17.3)	29 (23.4)	
Light diet	Yes	84 (62.7)	106 (85.5)	<0.001^∗∗^
	No	50 (37.3)	18 (14.5)	
*Clinical outcomes*				
BMI (kg/m^2^)		24.35 ± 3.15	24.18 ± 3.41	0.76
WC (cm)		83.14 ± 8.75	82.07 ± 9.33	0.35
FBG (mmol/L)		8.23 ± 3.44	7.90 ± 2.18	0.26
HbA1c (%)		7.17 ± 1.32	7.91 ± 1.77	0.19
SBP (mmHg)		128.99 ± 11.06	131.89 ± 13.89	0.20
DBP (mmHg)		75.06 ± 7.21	76.11 ± 7.33	0.18
Total cholesterol (mmol/L)		4.69 ± 1.02	4.74 ± 1.05	0.88
HDL-c (mmol/L)		1.24 ± 0.32	1.24 ± 0.34	0.81
LDL-c (mmol/L)		2.74 ± 0.86	2.76 ± 0.96	0.70
Triglyceride (mmol/L)		1.81 ± 1.12	1.90 ± 1.17	0.71

Data are presented as *n* (%) or means ± SD; values based on independent sample *t*-test for continuous variables and chi-square test for categorical variables to examine differences between the two groups (^∗^*P* < 0.05, ^∗∗^*P* < 0.01). BMI: body mass index; WC: waist circumference; FBG: fasting blood glucose; HbA1c: glycosylated hemoglobin A1c; SBP: systolic blood pressure; DBP: diastolic blood pressure; HDL-c: high-density lipoprotein cholesterol; LDL-c: low-density lipoprotein cholesterol.

**(b):** 

Characteristics	Intervention (*N* = 134)	Control (*N* = 124)	*P* value
*SF-36 scores*			
PF	67.90 ± 22.20	67.84 ± 25.39	0.99
RP	75.00 ± 38.78	66.73 ± 43.09	0.11
BP	78.61 ± 20.05	82.97 ± 19.24	0.08
GH	44.84 ± 17.11	44.85 ± 16.34	0.99
VT	46.26 ± 14.48	46.61 ± 15.37	0.85
SF	85.63 ± 20.25	76.11 ± 19.19	<0.001^∗∗^
RE	85.82 ± 33.55	74.73 ± 40.85	0.018^∗^
MH	59.41 ± 20.17	60.69 ± 20.63	0.61
PCS	48.34 ± 12.62	44.91 ± 14.30	0.48
MCS	43.86 ± 8.46	42.86 ± 9.56	0.08

Data are presented as means ± SD; values based on independent sample *t*-test for continuous variables to examine differences between the two groups (^∗^*P* < 0.05, ^∗∗^*P* < 0.01). SF-36: the Short Form 36; PF: physical functioning; RP: role limitations due to physical problems; BP: bodily pain; GH: general health; VT: vitality; SF: social functioning; RE: role limitations due to emotional problems; MH: mental health; PCS: physical component summary; MCS: mental component summary.

**Table 2:** Binary logistic regression analyses of health behavior outcomes in a 9-month group randomized experimental study in China, 2009-2010.

	Frequent smoker		Frequent drinker		Frequent exercise		Light diet	
	*N*	OR (95% CI)	*N*	OR (95% CI)	*N*	OR (95% CI)	*N*	OR (95% CI)
Intervention^a^	134	0.29 (0.05, 1.84)	134	0.07 (0.01, 0.75)^∗^	132	2.92 (1.18, 7.25)^∗^	134	4.30 (1.49, 12.43)^∗^
Age		0.97 (0.88, 1.08)		1.01 (0.91, 1.13)		0.98 (0.94, 1.03)		0.94 (0.89, 0.99)^∗^
Female^b^	148	0.07 (0.004, 1.05)	148	0.21 (0.02, 2.95)	146	2.04 (0.84, 4.98)	148	1.76 (0.69, 4.47)
Single, widowed, or divorced^c^	46	2.66 (0.21, 34.30)	46	1.37 (0.04, 49.15)	46	0.89 (0.21, 2.69)	46	0.68 (0.19, 2.37)
Junior middle school or higher^d^	153	0.19 (0.03, 1.23)	153	0.27 (0.04, 1.91)	153	1.31 (0.54, 3.17)	153	1.13 (0.44, 2.91)
Household income ≥ 60000 Yuan^e^	47	1.45 (0.22, 9.66)	47	3.40 (0.26, 44.81)	47	3.69 (0.93, 14.70)	47	3.14 (0.65, 15.23)
Retirement^f^	242	0.21 (0.01, 4.22)	242	0.81 (0.02, 29.42)	240	1.29 (0.23, 7.11)	242	0.80 (0.13, 5.04)
Diabetes duration ≥ 5 years^g^	199	0.61 (0.10, 3.79)	199	0.42 (0.04, 4.56)	197	1.59 (0.60, 4.25)	199	0.81 (0.26, 2.52)
Insulin use^h^	32	1.18 (0.15, 9.23)	32	0.84 (0.04, 18.44)	32	0.67 (0.21, 2.15)	32	0.76 (0.20, 2.89)
No/unknown other chronic disease^i^	212	0.23 (0.03, 1.85)	212	1.98 (0.19, 20.33)	210	0.66 (0.22, 1.98)	212	1.15 (0.36, 3.66)
Health behaviors at baseline (no)^j^	243	0.01 (0.001, 0.06)^∗^	238	0.01 (0.00, 0.06)^∗^	52	0.15 (0.06, 0.34)^∗^	68	0.42 (0.15, 1.17)

Binary logistic regression analysis was used to analyze the association between health behaviors (yes/no) and independent variables including group, health behaviors (yes/no) at baseline, and individual characteristics (age, gender, marital status, educational level, household income, employment level, diabetes duration, diabetes medication, and diagnosis of other chronic disease), respectively. The levels of association were expressed as odds ratios (OR), and we calculated their 95% confidence intervals (CI). Frequent smoker: self-reported smoking one or more cigarettes a day; frequent drinker: self-reported drinking at least once a week, with an average intake of 25 g pure alcohol per day or above; frequent exercise: self-reported physical activity at least once a week; light diet: self-reported low-fat diet. ^a^Reference: control group; ^b^Reference: male; ^c^Reference: married or cohabiting; ^d^Reference: elementary school or lower; ^e^Reference: <60000 Yuan; ^f^Reference: employee/self-employed or housework; ^g^Reference: 1-5 years; ^h^Reference: noninsulin; ^i^Reference: yes; ^j^Reference: frequent smoker (yes); frequent drinker (yes); frequent exercise (yes); light diet (yes). ^∗^Indicate a statistically significant association.

**Table 3:** Comparison of clinical outcomes between the intervention and control groups from baseline to postintervention in a 9-month group randomized experimental study in China, 2009-2010.

	Intervention (*N* = 134)			Control (*N* = 124)			Adjusted change between groups, mean (95% CI)^∗^	Adjusted *P* value^Δ^
	Baseline	Follow-up	*P* value^†^	Baseline	Follow-up	*P* value^†^		
BMI (kg/m^2^)	24.35 ± 3.15	23.14 ± 3.26	0.14	24.18 ± 3.41	24.69 ± 3.20	0.004	-0.36 (-1.11, 0.40)	0.35
WC (cm)	83.14 ± 8.75	79.66 ± 6.30	<0.001	81.07 ± 9.33	80.68 ± 9.71	0.15	-0.26 (-1.77, 1.25)	0.73
FBG (mmol/L)	8.23 ± 3.44	6.51 ± 1.92	<0.001	7.90 ± 2.18	6.61 ± 2.16	<0.001	0.10 (-0.45, 0.64)	0.73
HbA1c (%)	7.17 ± 1.32	6.60 ± 0.96	<0.001	7.91 ± 1.77	7.45 ± 3.06	0.28	0.21 (-0.38, 0.77)	0.08
SBP (mmHg)	128.99 ± 11.06	129.05 ± 8.97	0.95	131.89 ± 13.89	131.47 ± 12.06	0.77	-1.39 (-3.80, 1.01)	0.26
DBP (mmHg)	75.06 ± 7.21	73.20 ± 5.94	0.006	76.11 ± 7.33	73.51 ± 7.32	0.003	-1.50 (-3.40, 0.39)	0.12
Total cholesterol (mmol/L)	4.69 ± 1.02	4.87 ± 0.99	0.10	4.74 ± 1.05	4.76 ± 1.24	0.90	-0.08 (-0.31, 0.16)	0.54
HDL-c (mmol/L)	1.24 ± 0.32	1.31 ± 0.35	0.08	1.24 ± 0.34	1.22 ± 0.32	0.66	0.02 (-0.07, 0.10)	0.71
LDL-c (mmol/L)	2.74 ± 0.86	2.80 ± 0.77	0.60	2.76 ± 0.96	2.90 ± 1.0	0.13	-0.06 (-0.30, 0.18)	0.62
Triglyceride (mmol/L)	1.81 ± 1.12	1.72 ± 1.14	0.46	1.90 ± 1.17	1.79 ± 1.11	0.34	-0.05 (-0.31, 0.21)	0.69

Data are presented as means ± SD; ^†^*P* values based on paired *t*-tests for within-group changes from baseline to postintervention; ^Δ^the effect of group was adjusted for patients' age, gender, marital status, educational level, household income, employment status, diabetes duration, diabetes medication, and diagnosis of other chronic disease. ^∗^Value interpretable in relation to the intervention group: a negative value indicates greater negative change, and a positive value indicates greater positive change in the intervention group compared with control subjects.

**Table 4:** Comparison of the SF-36 scores between the intervention and control groups from baseline to postintervention in a 9-month group randomized experimental study in China, 2009-2010.

	Intervention (*N* = 134)			Control (*N* = 124)			Adjusted change between groups, mean (95% CI)^∗^	Adjusted *P* value^Δ^
	Baseline	Follow-up	*P* value^†^	Baseline	Follow-up	*P* value^†^		
*SF-36* *scale/summary score*								
PF	67.90 ± 22.20	76.45 ± 24.42	<0.001	67.84 ± 25.39	66.38 ± 25.09	0.50	1.66 (-2.57, 5.901)	0.44
RP	75.00 ± 38.78	91.23 ± 26.42	<0.001	66.73 ± 43.09	72.98 ± 40.15	0.19	9.97 (3.33, 16.60)	0.003
BP	78.61 ± 20.05	78.44 ± 19.54	0.92	82.97 ± 19.24	75.81 ± 21.21	<0.001	-1.22 (-5.24, 2.80)	0.55
GH	44.84 ± 17.11	42.87 ± 15.80	0.17	44.85 ± 16.34	40.32 ± 16.40	<0.001	0.04 (-3.2, 3.29)	0.98
VT	46.26 ± 14.48	42.17 ± 12.42	0.002	46.61 ± 15.37	50.08 ± 13.97	0.03	-5.43 (-7.98, -2.89)	<0.001
SF	85.63 ± 20.25	84.79 ± 19.83	0.57	76.11 ± 19.19	80.85 ± 20.31	0.02	6.50 (2.37, 10.64)	0.002
RE	85.82 ± 33.55	96.27 ± 19.02	<0.001	74.73 ± 40.85	84.14 ± 35.17	0.04	8.06 (2.15, 13.96)	0.008
MH	59.41 ± 20.17	59.20 ± 16.87	0.90	60.69 ± 20.63	57.19 ± 24.23	0.06	4.55 (-3.63, 4.54)	0.83
PCS	48.34 ± 12.62	52.31 ± 8.41	<0.001	44.91 ± 14.30	47.05 ± 11.25	0.14	3.31 (1.22, 5.39)	0.002
MCS	43.86 ± 8.46	40.98 ± 7.34	<0.001	42.86 ± 9.56	44.90 ± 8.62	0.02	-1.30 (-2.91, 0.31)	0.11

Data are presented as means ± SD; ^†^*P* values based on paired *t*-tests for within-group changes from baseline to postintervention; ^Δ^the effect of group was adjusted for patients' age, gender, marital status, educational level, household income, employment status, diabetes duration, diabetes medication, and diagnosis of other chronic disease. ^∗^Value interpretable in relation to the intervention group: a negative value indicates greater negative change, and a positive value indicates greater positive change in the intervention group compared with control subjects.

## Data Availability

The data used to support the findings of this study are available from the corresponding author upon request.
